# Granulocyte-Colony-Stimulating Factor Effectively Shortens Recovery Duration in Anti-Thyroid-Drug-Induced Agranulocytosis: A Systematic Review and Meta-Analysis

**DOI:** 10.3389/fendo.2019.00789

**Published:** 2019-11-22

**Authors:** Yonghui Wang, Xiaoying Li, Qian Yang, Wei Wang, Yanan Zhang, Jun Liu, Liang Zheng, Bingbing Zha

**Affiliations:** ^1^Department of Endocrinology, Fifth People's Hospital of Shanghai Fudan University, Shanghai, China; ^2^Department of Geriatrics, Xinhua Hospital of Shanghai Jiaotong University, School of Medicine, Shanghai, China; ^3^Research Center for Translational Medicine, Shanghai East Hospital, Tongji University School of Medicine, Shanghai, China

**Keywords:** agranulocytosis, ATD, G-CSF, hyperthyroidism, treatment

## Abstract

**Background and aim:** Granulocyte-colony-stimulating factor (G-CSF) is highly beneficial as a general treatment for anti-thyroid drug (ATD)-induced agranulocytosis. This meta-analysis aimed to assess the clinical effects of G-CSF and non-G-CSF on recovery duration in patients with ATD-induced agranulocytosis by analyzing the overall clinical outcomes.

**Methods:** The PubMed, Embase, Ovid, Cochrane, Google Scholar, China National Knowledge Infrastructure (CNKI) databases were searched for published studies from 1900 to 2018. No language restriction was implemented.

**Results:** This meta-analysis included 10 published retrospective studies and one prospective study. Data were obtained from 11 trials (474 patients: 247 with G-CSF and 227 with non-G-CSF treatment). Compared with the non-G-CSF group, the G-CSF group presented shorter recovery duration [weighted mean difference (WMD) = −3.04 days, 95% confidence interval (95% CI): −4.38 to −1.69 (*Z* = 4.43 *P* = 0.000)]. However, the recovery duration varied across regions and recovery criteria. Asian patients achieved significant clinical outcomes [WMD = −3.16 days (95% CI: −4.58 to −1.74, *P* = 0.000)] compared with European and South American patients [WMD = −2.19 days (95% CI: −7.38 to 3.01, *P* = 0.409)]. Also, according to various recovery criteria, a duration of granulocyte count increase of more than 1.5 or 1.0 × 10^9^/L [WMD = −3.50 days (95% CI: −4.82 to −2.18*, P* = 0.000)] revealed a better treatment effect.

**Conclusion:** G-CSF can significantly shorten the recovery duration in patients with ATD-induced agranulocytosis.

## Introduction

Hyperthyroidism is caused by Graves' disease (GD), autonomously functioning thyroid adenoma, and so forth. It occurs in 12 out of 1,000 people, and 90% of patients are diagnosed with GD (1). Anti-thyroid drugs (ATDs) include propylthiouracil (PTU), methimazole (MMI), and carbimazole (2, 3). Patients who are not eligible for surgery or radioactive iodine therapy can be treated with ATD therapy (4). However, it has minor side effects, such as skin rash, mild hepatic dysfunction, and arthralgia, and rare but severe side effects, such as aplastic anemia (AA), agranulocytosis, myeloperoxidase antineutrophil cytoplasmic antibody–related vasculitis, and hepatotoxicity (2, 3). AA is a severe type of hematological damage in which bone marrow stops producing blood cells, resulting in a higher risk of uncontrolled bleeding and infection (5). Although AA seems to be more severe than agranulocytosis, the present study did not focus on it due to a lack of adequate clinical information (6). In fact, hematological damage is caused by not only ATDs but also by GD. Further, 19 cases of pancytopenia were reported to be unrelated to ATD. The counts of white cells, red blood cells, and platelets dropped sharply without applying ATDs (7). The ATD-unrelated cases indicated that the thyroid hormone had a direct impact on hematopoiesis. This study focused on ATD-induced agranulocytosis, which is more common in clinic. Agranulocytosis has been defined as a granulocyte count of <0.5 × 10^9^/L (8). ATD-induced agranulocytosis occurs in 0.1–0.5% of patients with hyperthyroidism, mainly in the first 4 months after the initiation of ATD treatment (2, 9, 10). The typical symptoms are fever, sore throat, and infection (11). ATD treatment should be discontinued as soon as agranulocytosis is diagnosed. The basic treatments for ATD-induced agranulocytosis are G-CSF, steroids, antibiotics, and other drugs that increase the leukocyte count (2, 11).

Nevertheless, the treatment of ATD-induced agranulocytosis has been controversial. Many retrospective studies have focused on ATD-induced agranulocytosis, and some concluded that G-CSF could shorten the recovery duration. However, some indicated that there was no statistically significant difference between the recovery duration in G-CSF and non-G-CSF groups. In 2006, the American Society of Clinical Oncology (ASCO) provided updated recommendations for patients with non-cancer- or non-chemotherapy-induced agranulocytosis. According to this guideline, patients with high-risk features, such as prolonged and profound agranulocytosis, an age of more than 65 years, uncontrolled primary diseases, pneumonia, hypertension, and multiorgan dysfunction, should take G-CSF as an adjunct treatment (12, 13). However, treating these patients based on the same guideline is not appropriate, because many other drugs besides ATDs induce agranulocytosis, with various mechanisms. This systematic review and meta-analysis was conducted to summarize the published studies evaluating the practical effects of G-CSF in ATD-induced agranulocytosis. The measurable outcome of G-CSF was the recovery duration: the period from the time when agranulocytosis was diagnosed to the time when the granulocyte count met the recovery criteria.

## Materials and Methods

This meta-analysis was performed based on the Preferred Reporting Items for Systematic Reviews and Meta-analysis (14) and the Meta-analysis of Observational Studies in Epidemiology (15).

### Data Sources and Search Strategy

A systematic literature review was performed based on the databases PubMed, Embase, Medline, Ovid, Cochrane, CNKI, and Google Scholar. The keywords used for the search were hyperthyroidism, GD, agranulocytosis, leukopenia, granulocyte colony-stimulating factor, and G-CSF. The search range was extended by combining the keywords with the following free words: low white blood cell count and granulocytopenia. The advanced search protocol was restricted to studies from 1900 to 2018, but the language was unrestricted. Two researchers, Yonghui Wang and Xiaoying Li, extracted the data independently.

### Study Selection and Eligibility Criteria

The inclusion criteria were as follows: (1) patients rigorously diagnosed with hyperthyroidism and agranulocytosis; (2) patients with ATD-induced agranulocytosis; (3) studies including both G-CSF and control groups; (4) recovery duration expressed as mean and standard deviation (SD), or median and range. The exclusion criteria were as follows: (1) studies without a control group; (2) patients from different case reports; (3) research enrolling few patients or fail to provide mean and SD, or median and range; (4) no statement that the granulocyte count was normal before hyperthyroidism; (5) abstracts, reviews, and case reports; and (6) agranulocytosis induced by other drugs.

### Data Extraction and Quality Assessment

The information was extracted from the included studies using a standard form by two reviewers (Yonghui Wang and Xiaoying Li) independently. Any disagreement was resolved by discussion with a third reviewer (Liang Zheng). The standard form included the following variables: author, publication year, journal, research period, patients' races, drug used in the studies, dosage of drugs, mean age of patients, number of female and male patients, dosage of G-CSF, number of patients, follow-up period, and mean and SD of G-CSF and non-G-CSF groups. The quality of the non-randomized studies was assessed using the Newcastle–Ottawa Scale with the following items: patient selection, comparability, and assessment of outcome. The studies scoring five or more stars were considered to be of high quality. Also, a checklist with 4–11 items, which was recommended by the Agency for Healthcare Research and Quality (AHRQ), was used as an alternative tool to assess the quality of studies. In this form, an item scored “0,” if the answer was “No” or “Not clear,” and “1,” if the answer was “Yes.” Scores of 8–11 indicated high quality; scores of 4–7 indicated moderate quality; and scores of 0–3 indicated low quality. Potential publication bias was assessed using funnel plots and was also evaluated using Begg's test. Publication bias was not existent when the *P*-value was more than 0.1 assessed by funnel plots and Begg's test.

### Statistical Analysis

Stata software version 14 (Stata Corp., TX, USA) was used to perform the meta-analysis. For those only providing the median and range of recovery duration, the mean and SD were calculated as recommended by Stela Pudar Hozo (16). Heterogeneity was assessed using the χ^2^ and *I*^2^ statistics. When the *P-*value was < 0.05 or the *I*^2^ value was <50%, the heterogeneity was acceptable and provided proof that the fixed-effects model could be used to calculate the pooled effect. A forest plot was used to summarize the effect of G-CSF compared with non-G-CSF in terms of shortened recovery duration and to estimate the 95% confidence interval. Meta-regression analysis was performed to identify the potential source of bias. Sensitivity analyses were also conducted, excluding one study of the pooled effects to balance one study's influence with overall effects.

## Results

### Study Identification and Selection

**Figure 1:**
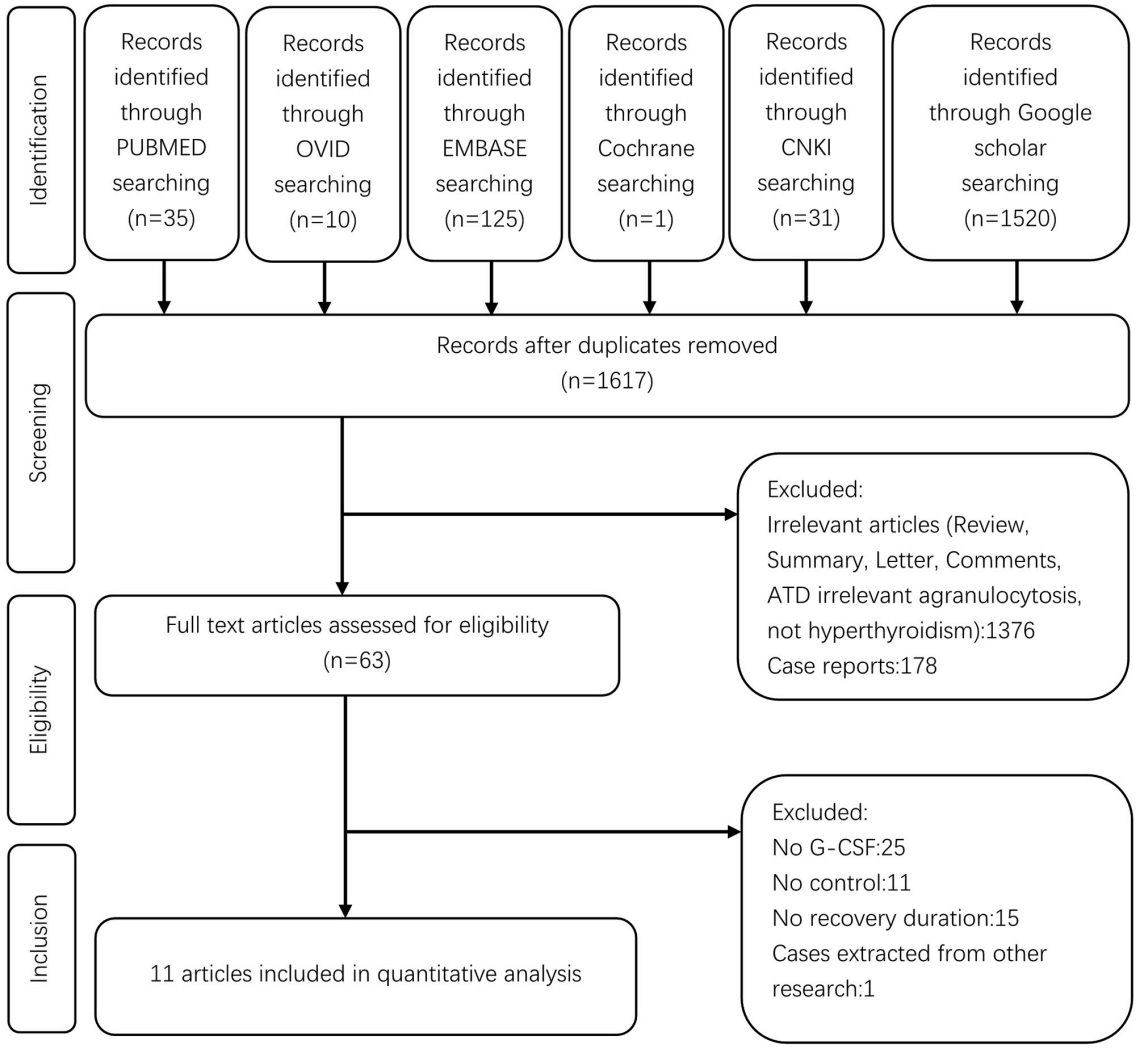
Flow diagram of the search strategy.

A flow chart for this meta-analysis is shown in Figure 1. A total of 1,722 studies were retrieved from the databases. After removing 105 duplicates by screening the titles, 1,617 studies were found to be relevant. Of these, 1,554 studies were excluded after reviewing the abstracts, and 63 full-text studies were screened in detail. Out of these 63 studies, 25 failed to provide information on G-CSF treatment, only 27/63 included patients with G-CSF treatment vs. those without G-CSF as the control group, 11 related studies failed to provide information on recovery duration, and one included cases from other studies. Finally, 11 studies were eligible for the meta-analysis.

### Characteristics of Included Studies

Of the 11 studies included, 10 were of a retrospective nature and one was a prospective study. The publication time ranged from 1993 to 2016. The participants came from different regions, including Asia, Europe, and South America. The medical records were retrieved from 1975 to 2016. Further, 247 patients received G-CSF after diagnosis with agranulocytosis and 227 received antibiotics, steroids, or no treatment. Five studies included patients with hyperthyroidism, revealing that the incidence of agranulocytosis in hyperthyroid patients ranged from 0.1 to 3.5%. One study did not clarify the definition of agranulocytosis, one study stated that the agranulocytosis was defined as an absolute granulocyte count of <2.0 × 10^9^/L, and nine studies clearly stated that agranulocytosis was diagnosed when the absolute granulocyte count was <0.5 × 10^9^/L. Eight of 11 studies mentioned the recovery criteria. Among these, five studies reported the recovery of patients with a granulocyte count reaching 0.5 × 10^9^/L, one study set the criteria to 1.0 × 10^9^/L and 1.5 × 10^9^/L, and four studies did not clarify the recovery criteria. All patients stopped taking ATDs when diagnosed with agranulocytosis. Only six studies reported the G-CSF doses of patients, which were 75, 300, 5, and 100–250 μg/day. The details of the included studies are shown in Table 1.

**Table 1:** Characteristics of the included studies.

	**Location**	**Year published**	**Number of Patients(F/M)**	**Age**	**Type Of ATD**	**ATD Dose**	**G-CSF Dose**	**Recovery Criteria**	**Research Period**	**G-CSF group**		**Control group**		**Follow-up**
										**Patients number**	**Recovery duration**	**Patients number**	**Recovery duration**	
Yang et al. (17)	China	2016	114 (104/10)	41.7 ±12.3	PTU MMI	22.9 ± 8.0 253.6 ±177.5	NR	>1.0 × 10^9^	2000–2015	93	12.7 ± 6	21	16.4 ± 10.6	19.6 (6–104)
Tajiri et al. (18)	Japan	2005	109 (103/6)	40.2 ±14.7	PTU MMI	25 ± 7.9 365.6 ±102.8	75 ug/d	>0.5 × 10^9^	1975–2001	19	5.5 ± 3.5	37	9.2 ± 4.4	>3 m
										15	2.3 ± 1.9	34	5.4 ± 4.3	
Fukata et al. (19)	Japan	1999	24 (23/1)	37 ± 21 [No G-CSF] 32 ± 14[G-CSF]	PTU MMI	24 270	100–250 ug/d	>0.5 × 10^9^	1990–1995	14	6.9 ± 3.8	10	7.6 ± 3.3	NR
Zelada et al. (20)	Peru	2016	29 (25/4)	34 (29–43)	PTU MMI	NR	NR	NR	2010–2016	12	9.5 ± 5.9	17	9 ± 5.2	NR
Clauna-Lumanta et al. (13)	Philipine	2016	28 (23/5)	28 (18–64) [no G-CSF] 39.5 (21–55)[G-CSF]	PTU MMI	NR	NR	NR	2005–2014	14	4.2 ± 2	14	10.2 ± 5.3	NR
Watanabe et al. (21)	Japan	2012	50 (47/3)	42 ± 17.9	PTU MMI	30 (5,30) 300(100,300)	NR	>0.5×10 ^9^	1983–2002	35	8 ± 4.2	8	10.7 ± 5	NR
Andrès et al. (22)	France	2001	20 (19/1)	62 ± 14.3	Carbimazole Benzylthiouracil	30 ± 12 100 ±37	300 ug/d	>1.5×10 ^9^	1985–2000	10	6.8 ± 4	10	11.6 ± 5	NR
Huang et al. (23)	Taiwan	2007	13 (10/3)	39.6 ± 10	PTU MMI Carbimazole	22.5 ± 7.5 300 ± 50 36.7 ±17	300 ug/d	>0.5×10 ^9^	1989–2003	3	9 ± 3.6	10	7 ± 3.2	>6 m
Sheng et al. (24)	Taiwan	1999	13 (10/3)	13.8 ± 17.8	MMI Carbimazole	15–30 30	5 ug/kg	>0.5 × 10 ^9^	1987–1997	6	8.4 ± 3.5	5	7.3 ± 5	NR
Tamai et al. (25)	Japan	1993	24/12000 (27/7)	38.4 ± 6.8	MMI	NR	75 ug/d	>1.0 × 10^9^	1979–1991	12	6.8 ± 1.2	11	10.1 ± 2.2	NR
Gao et al. (26)	China	2006	64 (49/15)	26.0 ± 8.4	PTU	200–600	NR	NR	2004–2005	14	4.2 ± 2.1	50	10.4 ± 3.6	NR

*G-CSF, Granulocyte colony-stimulating factor;MMI, methimazole;NR, not reported; PTU,propylthiouracil*.

### Study Quality

All studies were cohort studies except one, which was a prospective study. Hence, the quality prospective study was not assessed using the NOS scale or AHRQ form. Further, 36.4% (4/11) of the included studies were evaluated to be of high quality with more than five stars in the NOS scale, and 45.5% (5/11) were evaluated to be of moderate quality in the AHRQ form. The risk of bias of the included studies was low. As the control groups belonged to the same center, it was supposed that they came from the same community. The participants could moderately represent the G-CSF and non-G-CSF cohorts. Some studies failed to make a statement that agranulocytosis did not exist before hyperthyroidism, lacked comparability of the two groups in terms of age, sex ratio, and drug dose, or lacked follow-up to trace the later condition when considering the compatibility between G-CSF and non-G-CSF groups and outcomes of the studies. The diagnosis and characteristics of all patients were based on the hospital records, and therefore they were regarded as secure data. Most studies indicated that they excluded suspicious patients. Yet, most studies did not provide information on the exact follow-up period because they did not focus on the recurrence of agranulocytosis after the first treatment. Due to its low incidence, the value of studying recurrence was restricted. Hence, it was considered that the loss of follow-up would not produce bias.

### Primary Outcome

Eleven studies provided data on recovery duration depending on the application of G-CSF, except for one study, which had an elaborate design containing two subgroups: symptomatic and asymptomatic. As the differences in recovery duration in symptomatic and asymptomatic groups were statistically significant, they were segregated into two profiles.

**Figure 2:**
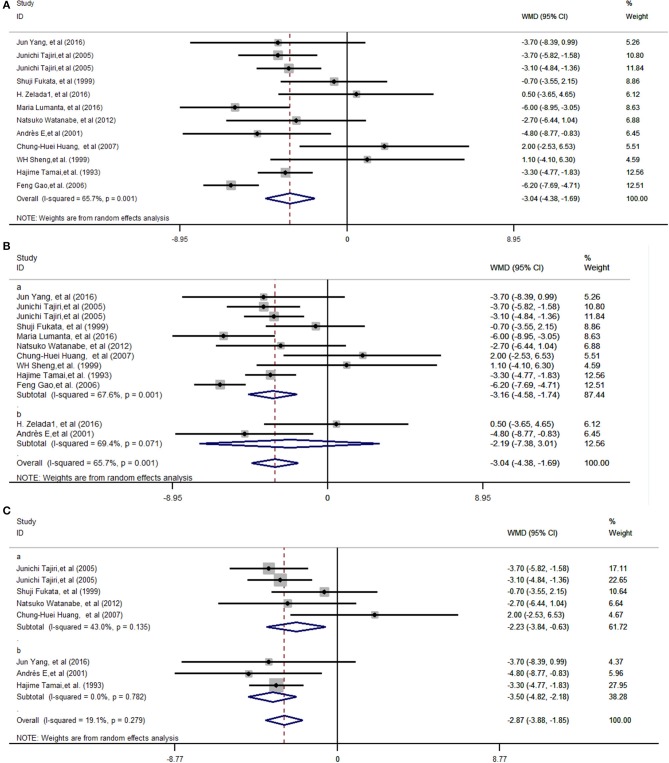
Forest plots depicting the recovery duration for ATD-induced agranulocytosis between G-CSF and non-G-CSF groups. Diamonds indicate the overall summary estimate (width of the diamonds represents the 95% CI); boxes indicate the weight of individual studies in the pooled analysis; dots indicate the WMD of each study; bars indicate the 95% CI of each study. It shows an overall pooled WMD of −3.04 days (95% CI = −4.38 to −1.69, *P* = 0.000) in the random-effects model, indicating that G-CSF can shorten the recovery duration of ATD-induced agranulocytosis **(A)**. The pooled effects in different regions, Asia **(B**a**)**, and in South America and Europe **(B**b**)**, were a WMD of −3.16 days (95% CI: −4.58 to −1.74, *P* = 0.000) and a WMD of −2.19 days (95% CI: −7.38 to 3.01, *P* = 0.409), respectively. The subgroup effects with different recovery criteria showed that when the granulocyte count was more than 0.5 × 10^9^/L **(C**a**)**, the WMD was −2.23 (95% CI: −3.84 to −0.63, *P* = 0.006), and when the criteria was a granulocyte count of more than 1.0 × 10^9^/L or 1.5 × 10^9^/L **(C**b**)**, the WMD was −3.50 days (95% CI: −4.82 to −2.18, *P* = 0.000). CI, Confidence interval; WMD, weighted mean difference.

The heterogeneity analysis showed that the data were heterogeneous (χ^2^ = 32.06 and *I*^2^ = 65.7%, *P* = 0.001) (Figure 2A). The random-effects model was used to calculate the total effect and subgroup effects. The pooled analyses shown in Figure 2A demonstrated that G-CSF was able to efficiently shorten the recovery duration [weighted mean difference (WMD) = −3.04 days, 95% confidence interval (95% CI): −4.38 to −1.69 (*Z* = 4.43 *P* = 0.000)].

As nine Asian studies and two studies from Europe and South America were enrolled, it was considered that the pooled effects were more typical in Asia. The subtotal effects indicated that the pooled effects in Asia (WMD = −3.16 days, 95% CI: −4.58 to −1.74, *P* = 0.000) (Figure 2Ba) were statistically significant. On the contrary, the pooled effects in Europe and South America (WMD = −2.19 days, 95% CI: −7.38 to 3.01, *P* = 0.409) proved that the mean recovery duration in the G-CSF group did not statistically significantly decrease compared with that in the non-G-CSF group (Figure 2Bb). Also, studies with different recovery criteria as the endpoint, except studies that did not define recovery criteria, were considered in two subgroups. For studies whose recovery criterion was a granulocyte count of more than 0.5 × 10^9^/L, WMD was −2.23 (95% CI: −3.84 to −0.63, *P* = 0.006) (Figure 2Ca). The recovery duration with a granulocyte count of more than 1.0 × 10^9^/L or 1.5 × 10^9^/L (Figure 2Cb) with WMD = −3.50 days (95% CI: −4.82 to −2.18, *P* = 0.000) presented larger differences between G-CSF and control groups compared with the criteria of a granulocyte count of more than 0.5 × 10^9^/L.

### Assessments of Bias

**Figure 3:**
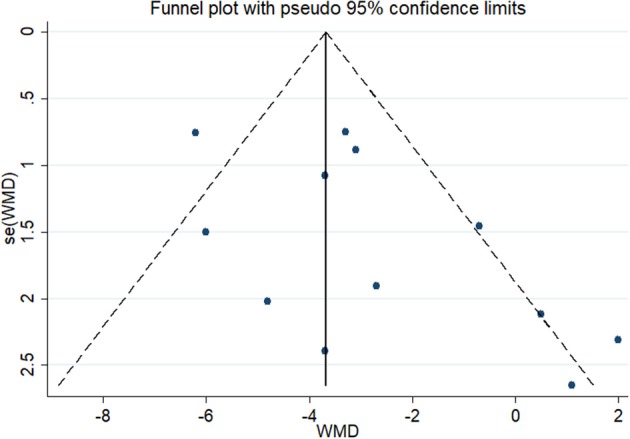
Funnel plot for publication bias.

In the test for publication bias, the Begg's funnel plot showed a symmetrical distribution, the Kendall score was 16 and the *Z* value was 1.03 (*P* = 0.304) (Figure 3) which indicated no statistical evidence of publication.

## Discussion

No previous meta-analysis has clarified the effect of G-CSF on agranulocytosis after hyperthyroidism. The guideline of the American Thyroid Association does not provide any recommendations to support or oppose the application of G-CSF in agranulocytosis after hyperthyroidism (4). The recommendation from the ASCO indicated that patients with afebrile neutropenia should not use G-CSF as an routine therapy, but for those febrile neutropenia patients with high risk factors should use G-CSF to avoid severe complication (12). A large number of reports and studies have focused on this medical problem, but it has been difficult to offer consistent advice. The only prospective study enrolling 20 patients suggested that G-CSF failed to shorten the recovery duration (19). However, in a single-center retrospective study involving more than 50,385 patients with GD, the recovery duration was significantly shorter in the G-CSF treatment group than in the non-G-CSF treatment group (21). Randomized controlled studies seemed more convincing than retrospective studies, but a large number of studies were retrospective. Thus, the published studies were summarized, and statistics-based advice was provided.

The present study demonstrated that G-CSF treatment was effective in ATD-induced agranulocytosis. The studies included were screened, and it was ascertained whether agranulocytosis occurred before ATD treatment. Therefore, it was believed that agranulocytosis after hyperthyroidism was associated with ATD. G-CSF could clearly shorten recovery duration compared with non-G-CSF treatment in which antibiotics and steroids were taken instead. In studies with patients in the non-G-CSF group taking antibiotics and steroids, the effect of G-CSF was also more significant. Hence, G-CSF was considered to be more effective than antibiotics or steroids. The exact differences among G-CSF, antibiotics, and steroids were not elaborated upon in the present study. Only six death cases were reported in three studies (20, 21, 24), of which four (1.76%) patients took non-G-CSF therapy and two patients (0.81%) who died of uncontrolled infection took G-CSF. This implied that G-CSF treatment could decrease the mortality in patients with ATD-induced agranulocytosis.

A subgroup analysis was performed considering that most of the studies included were performed in Asia, and recovery was defined as a granulocyte count of more than 0.5 × 10^9^/L. Further, 91.1% (225) of Asian patients took G-CSF, and of 88.1% (200) Asian patients did not take G-CSF after agranulocytosis. The subtotal results indicated that Asian patients presented convincing treatment response with obvious statistical significance. On the contrary, the subgroup effect in Europe and South America was not in favor of a distinct effect in the G-CSF group. This meta-analysis did not conclude that G-CSF failed to shorten the recovery duration in Europe and South America because only two studies were included in this subgroup. Additionally, few pertinent studies were conducted in other regions, which unavoidably restricted the intention of this meta-analysis. Moreover, it was not concluded that the effect of G-CSF would be more significant in Asia or other regions, as GD varied with different diets and races. The main source of carnitine is the diet, especially red meat, poultry, fish, and dairy products (27–29). Mahmoud et al. concluded that GD and its treatment were associated with pronounced acyl chain length-dependent alteration in acylcarnitine levels (30). Carnitine is known to impair the access of thyroid hormone to the nucleus, thereby diminishing thyroid hormone activity (31, 32). In an observational pilot study, the symptomatology of patients with subclinical hyperthyroidism dropped significantly by applying L-carnitine and selenium without any significant modifications of their endocrine profile (33). Europeans and Americans were prone to take more dietary protein and fat containing a higher amount of carnitine (34, 35). It seemed reasonable that the higher level of carnitine in European white races would be favorable to reestablishing euthyroidism. However, other studies have also pointed out that the level of acylcarnitine was relatively unremarkable in thyroid diseases (36). Thus, whether carnitine would have an influence on ATD-induced agranulocytosis in different regions is still controversial. Moreover, some genetic determinants play a role in susceptibility to ATD-induced agranulocytosis. For instance, the estimated odds ratios of HLA-B^*^38-02 and HLA-DRB1^*^08:03 comparing effective allele carriers to non-carriers were 21.48 and 6.13, respectively (37). These two loci had higher frequencies in the Asian population than in Caucasians (38). Moreover, in European patients, HLA-B^*^27:05 and single-nucleotide polymorphisms on chromosome 6 affected ATD-induced agranulocytosis (39). It was difficult to judge which one was the most critical in various Asian and European vulnerable loci. In addition, given the fact that the overall frequency of ATD-induced agranulocytosis did not diverge in different ethnic groups, it was supposed that several factors might be involved in the pathogenesis of ATD-induced agranulocytosis.

Moreover, two studies defined recovery as a granulocyte count of more than 1.0 or 1.5 × 10^9^/L. The subtotal effect showed a larger difference between the G-CSF and non-G-CSF groups. This might, to some extent, be explained by the granulocyte count at the onset of agranulocytosis, uncontrolled chronic diseases, bone marrow condition, and G-CSF dose. For a granulocyte count of <0.1 × 10^9^/L, G-CSF seemed to be invalid in increasing the granulocyte count (18). At the same time, complications related to chronic diseases would delay treatment response. In the study by Tania Sarker, the patients took more than 3 weeks to recover after G-CSF treatment. This lag time to response seemed to suggest a degree of depletion of committed myeloid progenitors and, indeed, pluripotent stem cells. Bone marrow examination showed that the M:E ratio was <0.5. Hence, bone marrow recovery from agranulocytosis after G-CSF treatment was delayed (40). Furthermore, the older the patients, the less active the bone marrow. Therefore, an age of more than 70 years was a prognostic factor for agranulocytosis after hyperthyroidism (41). Onose et al. explored potential predictive factors for neutrophil count recovery in patients with thiamazole-induced agranulocytosis. They found that decreased monocyte and basophil counts at onset might recover late and require careful treatment (42).

The strength of the present investigation was the strict process used in the search strategy so that the studies included were relatively homogeneous. To provide convincing data, Tajiri's study was separated into two profiles. Each subgroup was treated as an individual study because of the higher baseline of granulocyte count in the asymptomatic group (0.320 × 10^9^ ± 0.134 × 10^9^/L) than in the symptomatic group (0.186 × 10^9^ ± 0.162 × 10^9^/L, *P* < 0.001) and the shorter recovery durations in the asymptomatic group (2.3 ± 1.0 days) than in the symptomatic group (5.5 ± 3.5 days, *P* < 0.001). In both groups, the recovery periods were dramatically shorter than those before the prescription of G-CSF. Importantly, this was the first meta-analysis to summarize the overall clinical effect of G-CSF on ATD-induced agranulocytosis.

## Limitations

Nevertheless, this study had inevitable limitations. First, the baseline of the patients was varied. At the same time, some studies missed detailed information, such as the granulocyte count at the onset of agranulocytosis, follow-up period, or G-CSF dose. If the granulocyte count at onset was much lower, it was supposed that the recovery duration would be longer. Second, the association of ATD dosage with the recovery period could not be analyzed. Few studies provided the mean and SD of ATD dosage. Hence, it was not possible to make a subgroup analysis according to the ATD dosage. Third, the G-CSF dosage would be another prominent factor vital to guiding the medical decision. Some studies involved the use of varied dosages for patients, while some did not even mention the dosage. Thus, the meta-analysis could not offer further advice on G-CSF dosage. Fourth, nine studies were from Asia and two were from other regions, which made the conclusion more dependable when applied to Asian patients. The research strategy was expanded to more databases, but only two studies from South America and Europe were eventually found to be eligible for inclusion. Last but not least, 9 out of 10 included studies were of retrospective nature, which might weaken the strength of this meta-analysis. Due to the low frequency of ATD-induced agranulocytosis, prospective studies take a long time. Hence, only one prospective study was found. All these limitations were objective and difficult to overcome. Further studies should be conducted to provide detailed information on the characteristics at onset and G-CSF dosage so as to optimize the findings of the present analysis.

## Conclusion

G-CSF could effectively shorten the recovery duration in patients with ATD-induced agranulocytosis compared with non-GCSF treatment.

## Data Availability Statement

All datasets generated for this study are included in the article/supplementary material.

## Author Contributions

YW and XL were involved in drafting the manuscript and made a contribution to conception and design. QY engaged in the analysis and interpretation of the data. WW and YZ were involved in draft revision. JL contributed to the acquisition of data. BZ and LZ gave final approval to the version to be published. All authors agreed to be accountable for all aspects of the study in ensuring that questions related to the accuracy and integrity of any part of the study were appropriately investigated and resolved.

### Conflict of Interest

The authors declare that the research was conducted in the absence of any commercial or financial relationships that could be construed as a potential conflict of interest.
